# Supplementary material for: Analysis of Drugs in Saliva of US Military Veterans Treated for Substance Use Disorders Using Supported Liquid Extraction and Surface-Enhanced Raman Spectral Analysis
Source: Molecules. 2023 Feb 21;28(5):2010. doi: 10.3390/molecules28052010 (PMC10004423; doi:10.3390/molecules28052010)
Supplement: Supplementary file 1 [file molecules-28-02010-s001.zip › molecules-2215610-supplementary.pdf]

molecules

Supplementary Materials: Analysis of drugs in saliva of USA military veterans treated for substance-related disorders using supported liquid extraction and surface-enhanced Raman spectral analysis

Stuart Farquharson, Chetan Shende, Jenelle Newcomb, Ismene L. Petrakis and Albert J. Arias.

Table S1. Subject demographics, saliva and urine sampling times, daily buprenorphine dose, uranalysis, and other drugs used.

Sub ID Number	Race	Gender	Age	Vet/ Non-Vet	Date of Saliva Sample	Time of Saliva Sample	Date Saliva Sample Sent for Analysis	Date of Urine Sample	Time of Urine Sample	Daily BUP Dose	Urinalysis	Drugs used within 4 days of collected sample
1	Caucasian	M	65	V	6/26/2018	3:45PM	6/29/2018	6/26/2018	2:45PM	24mg	Positive: BUP	BUP
2	Caucasian	M	65	V	6/26/2018	1:45PM	6/29/2018	6/26/2018	2:25PM	20mg	Positive: BUP, cannabinoids	BUP, marijuana
3	Caucasian	M	58	V	6/26/2018	1:35 PM	6/29/2018	6/26/2018	2:30PM	8mg	Positive: BUP, amphetamines, cannabinoids	BUP, klonopin, marijuana
4	Caucasian	M	59	V	6/26/2018	1:30PM	6/29/2018	6/26/2018	1:15PM	24mg	Positive: BUP	BUP
5	Caucasian	M	62	V	6/25/2018	12:00PM	6/29/2018	6/25/2018	10:00AM	20mg	Positive: BUP	BUP
6	Caucasian	M	45	V	6/25/2018	11:45AM	6/29/2018	6/25/2018	11:45AM	16mg	Positive: BUP	BUP
7	AA	M	63	V	6/25/2018	2:45PM	6/29/2018	6/25/2018	2:40PM	20mg	Positive: BUP	BUP
8	AA	M	58	V	6/25/2018	10:45 AM	6/29/2018	6/25/2018	10:45 AM	20mg	Positive: BUP	BUP
9	Hispanic	M	65	V	6/25/2018	9:40AM	6/29/2018	6/25/2018	9:00AM	16mg	Positive: BUP, cannabinoids	BUP, marijuana
10	Caucasian	M	42	V	6/25/2018	9:20AM	6/29/2018	6/25/2018	8:45AM	8mg	Positive: BUP, cannabinoids	BUP, marijuana
11	AA	M	63	V	6/25/2018	9:10AM	6/29/2018	6/25/2018	8:45AM	24mg	Positive: BUP	BUP
12	AA	M	59	V	6/22/2018	1:40PM	6/29/2018	6/222018	12:00PM	8mg	Positive: BUP, opiates, methadone	BUP, heroin
13	declined to state race	M	46	V	6/22/2018	12:26 PM	6/29/2018	6/22/2018	12:28PM	24mg	Positive: BUP, cannabinoids	BUP
14	Hispanic	M	60	V	6/21/2018	1:25PM	6/29/2018	6/21/2018		24mg	Positive: BUP	BUP
15	Caucasian	M	44	V	6/21/2018	12:25PM	6/29/2018	6/21/2018	11:30AM	8mg	Positive: BUP	BUP
16	Caucasian	M	44	V	6/21/2018	11:40AM	6/29/2018	6/21/2018		8mg (started day of sample)	Positive: opiates	BUP (started same day as
17	Caucasian	M	36	V	6/21/2018	11:35 AM	6/29/2018	6/21/2018	11:30AM	20mg	Positive: BUP, cocaine	BUP
18	AA	M	65	V	6/21/2018	10:40 AM	6/29/2018	6/21/2018	9:30 AM	8mg	Positive: BUP, opiates	BUP, heroin
19	Caucasian	M	30	V	6/21/2018	10:22 AM	6/29/2018	6/21/2018	10:25 AM	24mg	Positive: BUP	BUP
20	Caucasian	M	49	V	6/20/2018	1:55 PM	6/29/2018	6/20/2018	12:00 PM	12mg	Positive: BUP, cannabinoids, cocaine	BUP, marijuana, cocaine

molecules

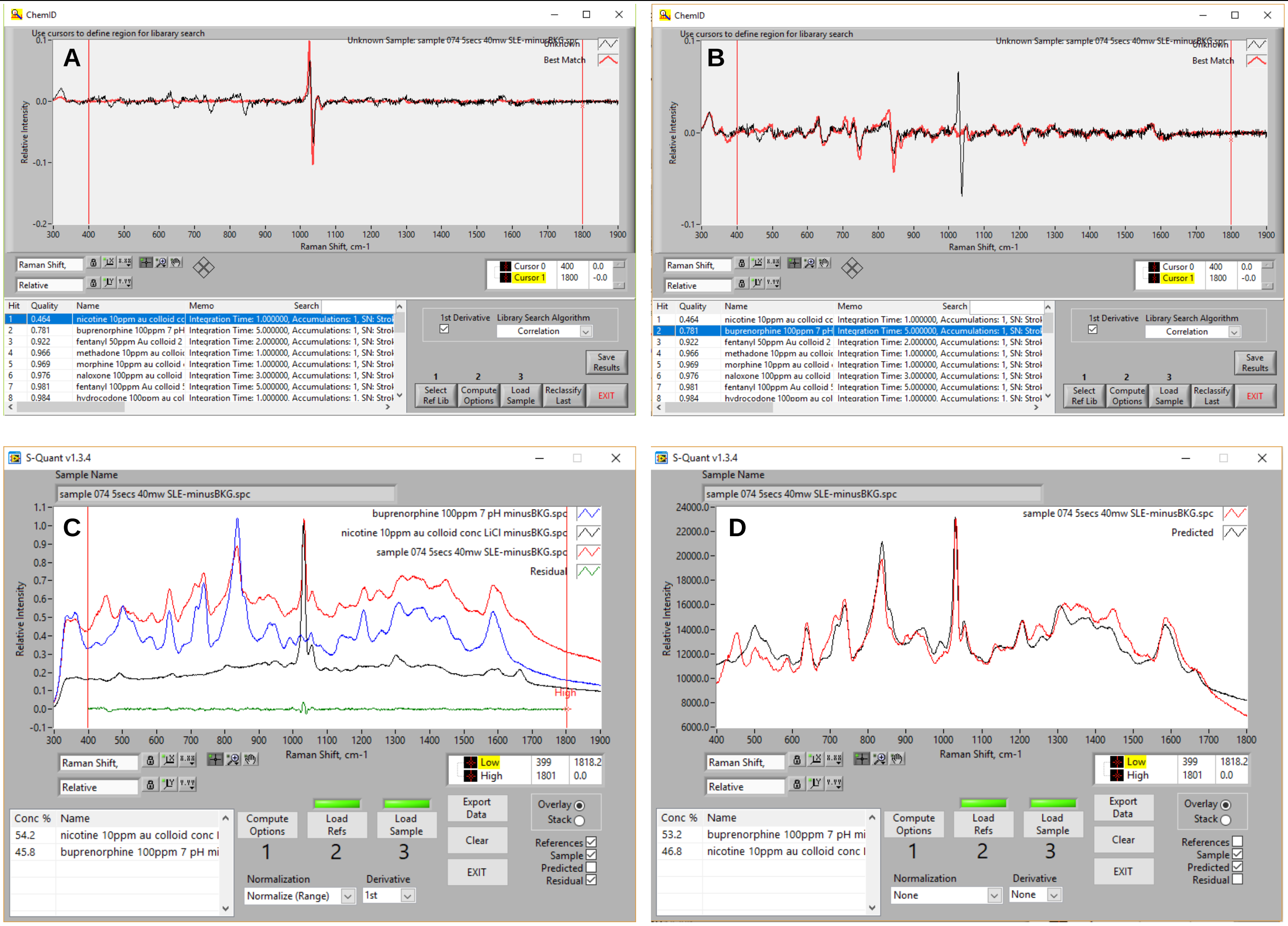

Figure S1. Images of Chem-ID software comparing Sample 1 to A) the first “Hit” nicotine, and B) the second “Hit” buprenorphine; and S-Quant software indicating the percent contribution for these two drugs using C) first derivative spectra and D) normal (untreated spectra). Spectral range used for analysis for all samples: 400 to 1800 cm^{-1}. Conditions: 100 mg/L sample in water, 40 mW at 785 nm, 1 sec acquisition.

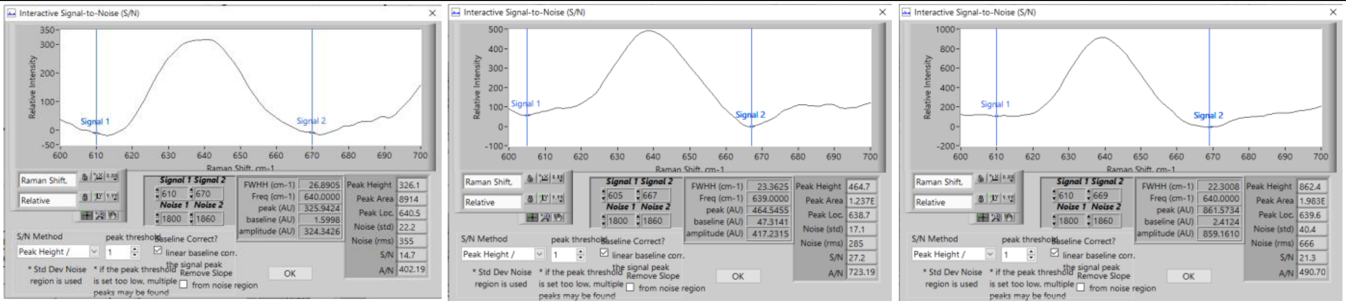

6.25 (326) 12.5 (465) 25 (862)

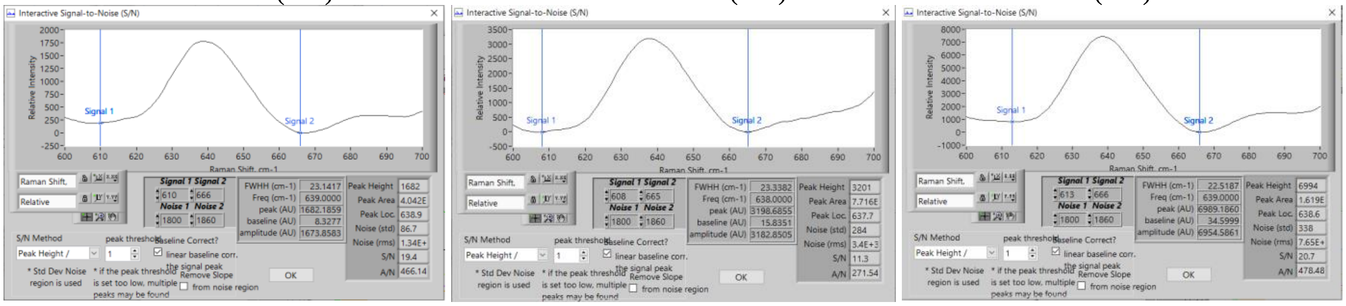

50 (1682) 100 (3201) 200 (6994)

Figure S2. Spectra of samples for buprenorphine concentration calibration curve. Peak heights indicated in parentheses. Conditions as in Figure S1.

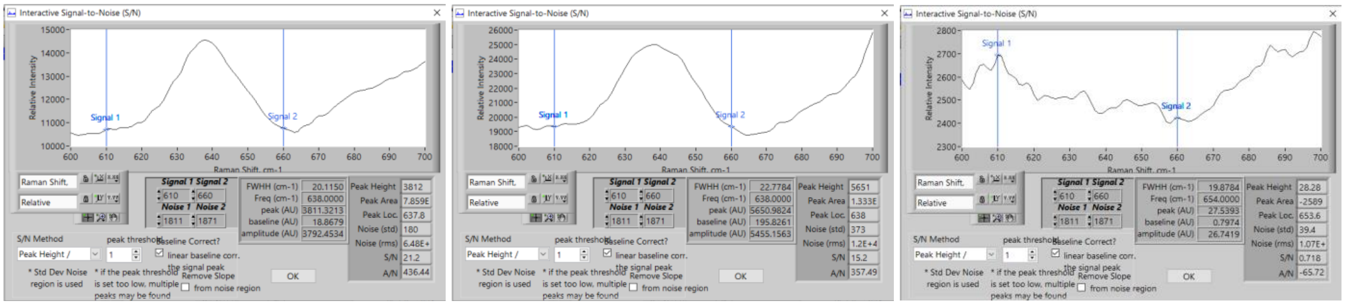

1 (3812) 2 (5651) 3 (28)

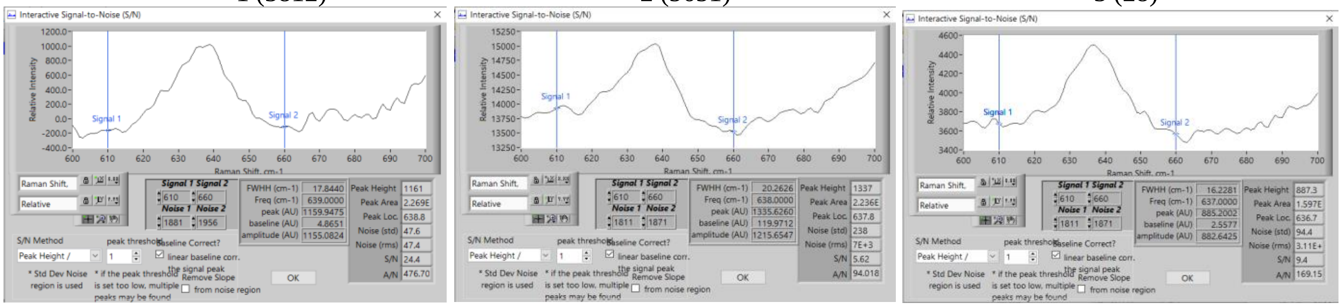

4 (1161) 5 (1337) 6 (887)

Figure S3a. Signal-to-noise software used to determine peak height with a baseline from 620 to 660 cm^{-1} for Samples 1-6. Peak heights indicated in parentheses. Conditions as in Figure S1.

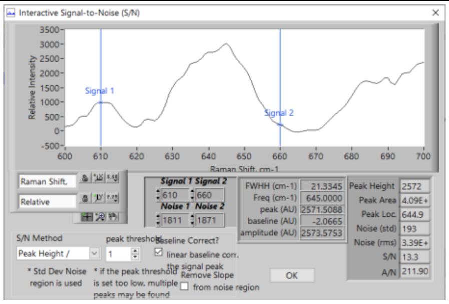

7 (2572)

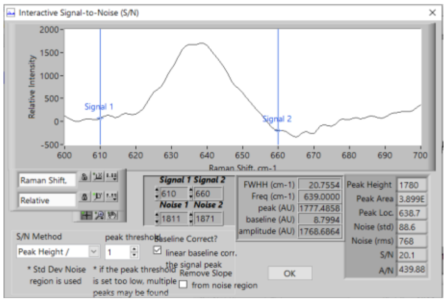

8 (1780)

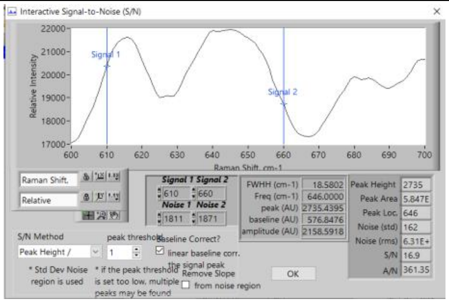

9 (2735)

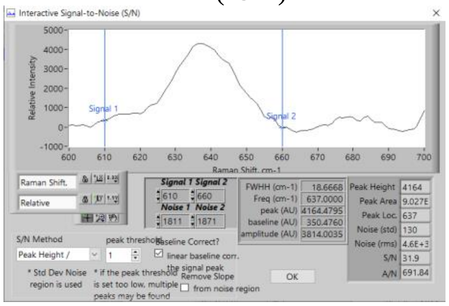

10 (4164)

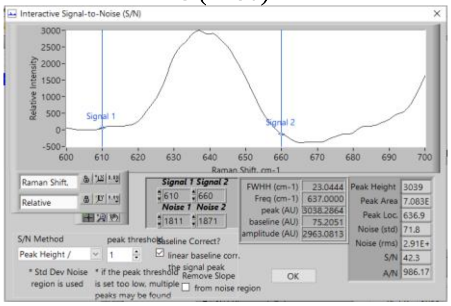

11 (3039)

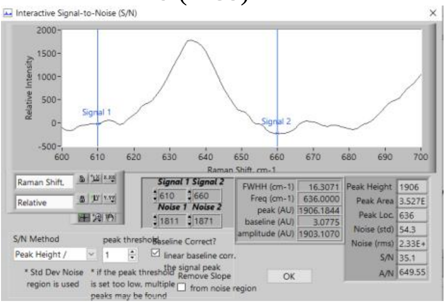

12 (1906)

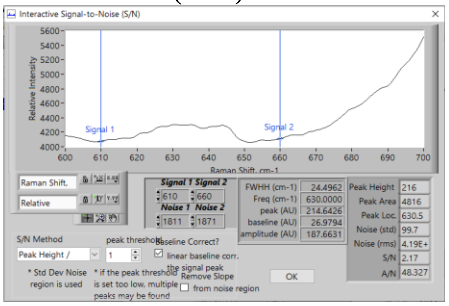

13 (216)

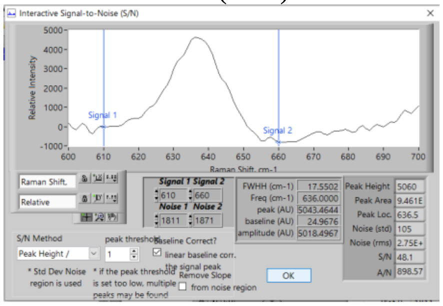

14 (5060)

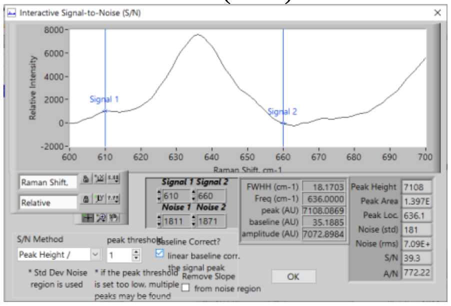

15 (7108)

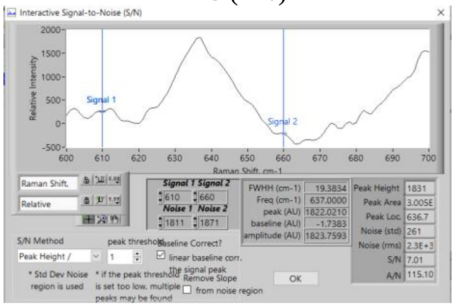

16 (1831)

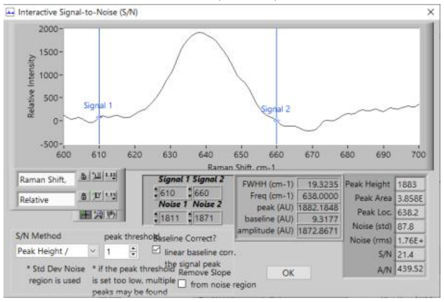

17 (1883)

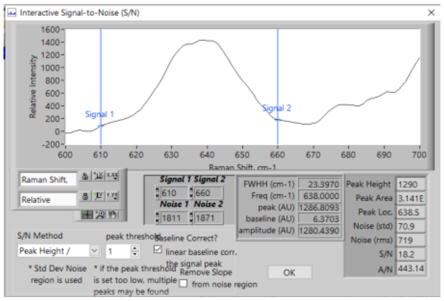

18 (1290)

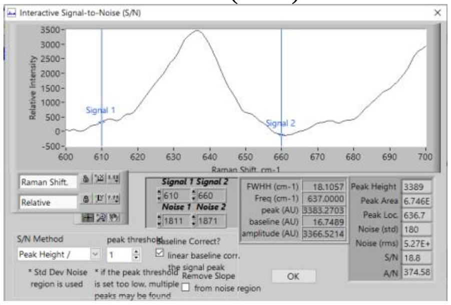

19 (3389)

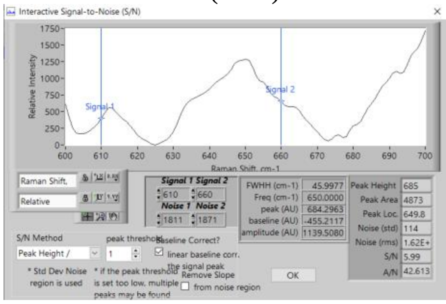

20 (685)

Figure S3b. Signal-to-noise software used to determine peak height with a baseline from 620 to 660 cm^{-1} for Samples 7-20. Peak heights indicated in parentheses. Conditions as in Figure S1.

molecules

MDPI

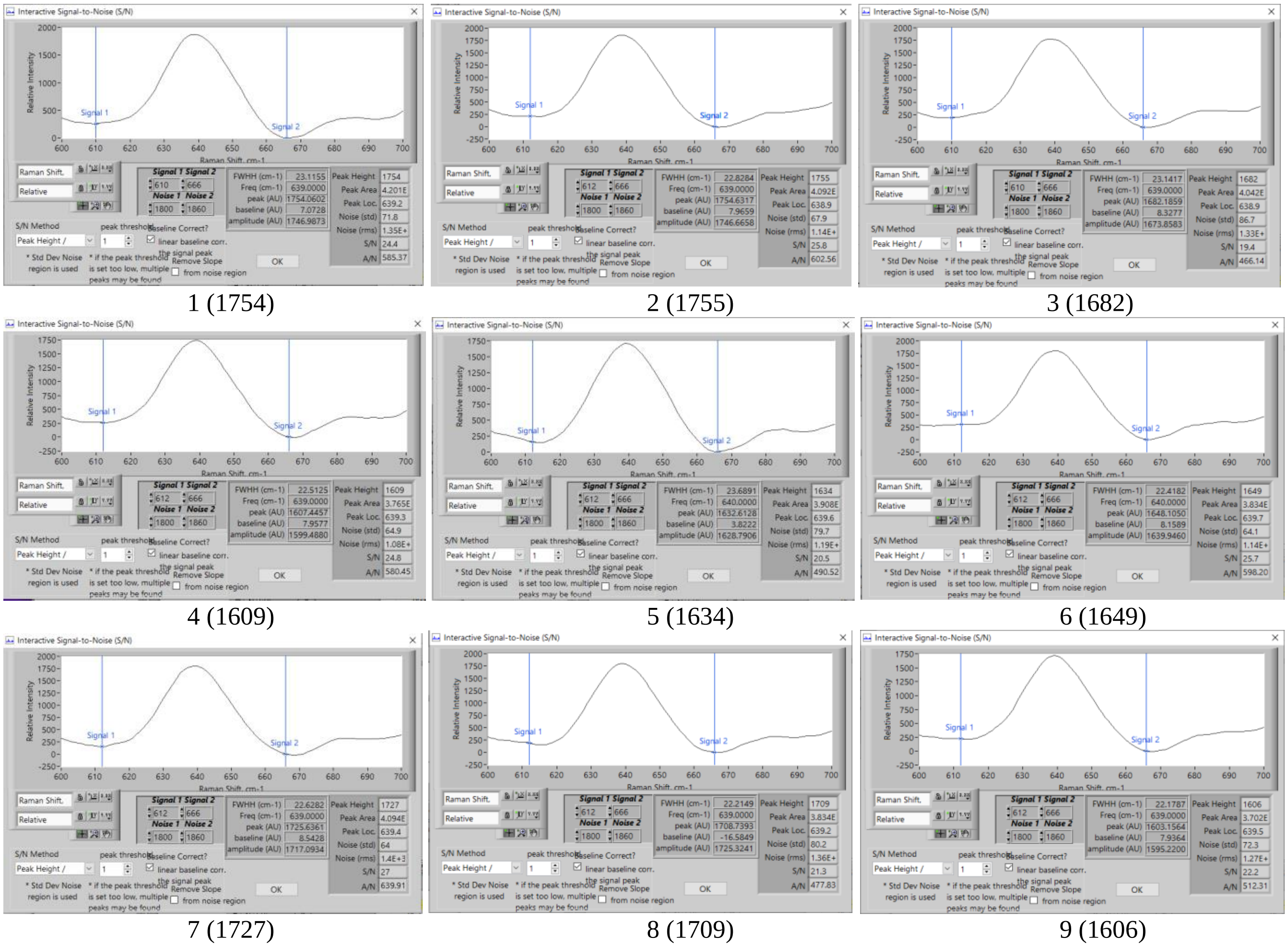

Figure S4. Nine repeat analyses for a 50 ng/mL prepared sample that included SLE preparation and SERS measurements. Peak heights indicated in parentheses. Conditions as in Figure S1.
